# High Intensity Interval and Endurance Training Have Opposing Effects on Markers of Heart Failure and Cardiac Remodeling in Hypertensive Rats

**DOI:** 10.1371/journal.pone.0121138

**Published:** 2015-03-24

**Authors:** Tanya M. Holloway, Darin Bloemberg, Mayne L. da Silva, Jeremy A. Simpson, Joe Quadrilatero, Lawrence L. Spriet

**Affiliations:** 1 Department of Human Health and Nutritional Sciences, University of Guelph, Guelph, Ontario, Canada; 2 Department of Kinesiology, University of Waterloo, Waterloo, Ontario, Canada; Emory University, UNITED STATES

## Abstract

There has been re-emerging interest and significant work dedicated to investigating the metabolic effects of high intensity interval training (HIIT) in recent years. HIIT is considered to be a time efficient alternative to classic endurance training (ET) that elicits similar metabolic responses in skeletal muscle. However, there is a lack of information on the impact of HIIT on cardiac muscle in disease. Therefore, we determined the efficacy of ET and HIIT to alter cardiac muscle characteristics involved in the development of diastolic dysfunction, such as ventricular hypertrophy, fibrosis and angiogenesis, in a well-established rodent model of hypertension-induced heart failure before the development of overt heart failure. ET decreased left ventricle fibrosis by ~40% (P < 0.05), and promoted a 20% (P<0.05) increase in the left ventricular capillary/fibre ratio, an increase in endothelial nitric oxide synthase protein (P<0.05), and a decrease in hypoxia inducible factor 1 alpha protein content (P<0.05). In contrast, HIIT did not decrease existing fibrosis, and HIIT animals displayed a 20% increase in left ventricular mass (P<0.05) and a 20% decrease in cross sectional area (P<0.05). HIIT also increased brain natriuretic peptide by 50% (P<0.05), in the absence of concomitant angiogenesis, strongly suggesting pathological cardiac remodeling. The current data support the longstanding belief in the effectiveness of ET in hypertension. However, HIIT promoted a pathological adaptation in the left ventricle in the presence of hypertension, highlighting the need for further research on the widespread effects of HIIT in the presence of disease.

## Introduction

Low aerobic capacity represents the greatest predictor of all-cause mortality, and is a clinically relevant parameter that is improved with chronic endurance exercise training [1]. The beneficial effects of endurance exercise training are multifaceted, affecting genetic programs in skeletal muscle that result in mitochondrial biogenesis, increased skeletal muscle capillarization, improved vascular compliance, and increased stroke volume and cardiac output [2–5]. As a result, chronic exercise training is a well-known primary and secondary intervention for various pathologies, including but not limited to heart failure (HF), hypertension, diabetes, as well as slowing the progression of aging [6–9].

While exercise training prevents disease and recovers the health of individuals, there is debate in the literature regarding the optimal intensity of exercise. Two types of exercise have largely been employed to elicit functional improvements in aerobic capacity, including classic endurance training (ET) and high-intensity interval training (HIIT). Acutely, in healthy subjects both types of exercise similarly activate signaling pathways, including p38 MAPK, CaMKII and AMPK [10]. Chronically, ET and HIIT result in comparable increases in skeletal muscle mitochondrial content, maximal activities of oxidative enzymes (e.g., citrate synthase, cytochrome c oxidase) the expression of plasma membrane transport proteins, glycogen content, and 24 hour post exercise energy expenditure [11–15]. These data suggest that HIIT may represent a time efficient clinically relevant tool to improve aerobic fitness in healthy individuals [16].

However, despite the wealth of data highlighting similar molecular and metabolic responses in skeletal muscle following different exercise training intensities in healthy individuals, intensity specific adaptations in the presence of disease, specifically within the diseased heart, remain to be elucidated. While ET increases capillarization and mitochondrial content, decreases fibrosis and prevents pathological hypertrophy in rodent models of HF, [17–19] the effect of HIIT on these molecular adaptations is unknown. Therefore, it remains to be determined if HIIT represents an optimal secondary prevention strategy in individuals with existing cardiovascular disease.

The ongoing debate regarding the effectiveness of ET and HIIT is further exemplified by the fact that ET, but not HIIT, consistently increases stroke volume and decreases heart rate (HR), decreasing the energetic demands of a beating heart in healthy individuals [20, 21]. The inconsistencies of the effects of HIIT in healthy populations makes the investigation of the use of HIIT in the presence of disease even more relevant, as the molecular signals and consequent adaptations are likely to be altered in disease states.

The effects of differing exercise intensities on diseased cardiac muscle are incompletely understood, therefore, we aimed to determine if ET and HIIT were comparable at altering various molecular responses in a rodent model of hypertension that is associated with the development of HF. Dahl/SS rats were chosen as a model of hypertension because of the rapid increase in blood pressure, pathological cardiac remodeling and mortality displayed after commencing a high sodium (HS) diet [22]. We hypothesized that both ET and HIIT would decrease left ventricular fibrosis, cross sectional area, and molecular markers of heart failure, and increase left ventricular capillarization similarly in Dahl/SS rats, providing clinical support for the use of HIIT.

## Methods

### Animals and experimental design

We aimed to determine if ET and HIIT had similar effects in preventing/exacerbating various molecular markers of HF. Male Dahl/SS rats were purchased from Charles River Laboratories (Saint-Constant, QC, CA) (8 weeks of age; n = 24). Dahl/SS rats fed a HS diet rapidly develop hypertension and display increased wall thickness and ventricular mass at 3 weeks, start to lose body weight after 5 weeks, with mortality beginning at 7 weeks [22]. We therefore chose to examine Dahl/SS rats after consuming a HS diet for 4 weeks when the only clinically relevant marker of disease was hypertension.

Rats were randomly assigned to receive a low sodium diet (LS, 0.3% NaCl chow, n = 6) or a HS diet (8% NaCl chow, n = 18) to induce the hypertensive phenotype. Diets were purchased through Research Diets (New Brunswick, NJ, USA), and were matched for caloric content and macronutrient composition. All animals fed the LS diet remained sedentary (LS-SED), while animals fed the HS diet were subdivided into three groups: sedentary (HS-SED, n = 6), classical endurance training (HS-ET, n = 6) and high intensity interval training (HS-HIIT, n = 6). The HS diets and exercise interventions commenced at the same time point (e.g., week 1). Animals were housed 1 per cage in a temperature-regulated room on a 12:12 hr light-dark cycle with water available *ad libitum*. This study was approved by the University of Guelph Animal Care Committee, and conforms to the guide for the care and use of laboratory animals published by the US National Institutes of Health.

### Treadmill Exercise

All rats were familiarized with a rodent treadmill (Columbus Instruments, Columbus, OH, USA) on at least 3 occasions (10 m/min, 0% grade, 10–15 min) before randomization. Exercise protocols were developed based on the American Physiological Society’s resource on exercise in animals, as well as from previous experience training rodents in our institution [23, 24]. The ET group trained 5 days/week for 4 weeks at progressively more challenging “moderate continuous” intensities. The HIIT groups also trained for 5 days/week for 4 weeks, however at progressively more challenging “intermittent high” intensities. Detailed protocols and average work done for both ET and HIIT can be found in Table 1. The average work intensity (Joules/min) [25] was ~50% higher in HIIT versus ET over the entire 4-week training intervention. Forty-eight hours after the last exercise bout, animals were anaesthetized with pentobarbital (100 mg/kg body weight) and the heart rapidly removed. After weighing the heart, a small sample from the LV was embedded in OCT for histochemical analysis, while the remainder of the LV was rapidly frozen in liquid nitrogen and stored at -80°C. The tibia was also removed, dissected and measured for length.

**Table 1:** Exercise Protocols and Work for HS-ET and HS-HIIT rats at weeks 1–4 of training.

		Week-1		Week-2		Week-3		Week-4	
Parameter		HS-ET	HS-HIIT	HS-ET	HS-HIIT	HS-ET	HS-HIIT	HS-ET	HS-HIIT
**Average Speed**	(m/min)	15	20	18	20	20	20	20	20
**Grade**	(%)	0	10	5	10	5	10	10	15
**Exercise Time**	(min/day)	30	8	45	15	45	23	45	23
**Average Work**	(Joules/min)	0.3	5	2	5	3	6	6	9

High sodium endurance training (HS-ET) and HS- high intensity interval training (HS-HIIT).

### Hemodynamics

Mean arterial blood pressure (MAP) was measured in conscious, restrained rats using a CODA® 2 tail-cuff system (Kent Scientific, Torrington, CT, USA) in a dark temperature-controlled room (22°C) in the morning. Rats were acclimatized on a minimum of three occasions prior to the study. On measurement days, conscious rats underwent 25 blood pressure measurements and MAP was averaged over the last 10 readings. HR was continuously monitored, with the corresponding last 10 measurement cycles averaged to calculate HR.

### Western blotting

Cardiac muscle (25–50 mg) was homogenized as previously reported [22] and 5μg of homogenate loaded for SDS-PAGE. Proteins were separated on a 6%, 7.5%, 10% or 12% resolving gel as required to optimize for MW separation, and transferred to polyvinylidene difluoride membrane (Roche, Laval, QC, CA). The following commercially available antibodies were used: total OXPHOS antibody cocktail (Abcam, Cambridge, MA, USA, ab110413, 1:500,), eNOS (Abcam, ab5589, 1:1000), VEGF (Abcam, ab46154, 1:1000), HIF1α (Abcam, ab463, 1:1000), alpha tubulin (Abcam, ab40742, 1:5000), muscle RING finger protein-1 (MuRF1; Santa Cruz Biotechnology, Dallas, TX, USA, sc-32920, 1:500), Muscle atrophy F-box (MAFbx; Santa Cruz, sc33782, 1:500), forkhead transcription factor-3a, Serine residue 253 (FOXO3a Ser253; Abcam, ab47285, 1:500), atrial natriuretic peptide (ANP; Abcam, ab180649, 1:500), BNP (Abcam, ab19645, 1:500) and beta-myosin heavy chain (β-MHC; Abcam, ab172967, 1:2000). All samples were detected from the same Western blot by cutting gels and transferring onto a single membrane to limit variability. Equal loading of protein was verified using Ponceau staining as well as constant alpha tubulin. All blots were quantified using enhanced chemiluminescence (Perkin Elmer, Woodbridge, ON, CA) and quantified by densitometry (Alpha Innotech Fluorchem HD2, Fisher Scientific, Ottawa, ON, CA).

### Citrate synthase activity

Citrate synthase (CS) activity was assayed in homogenates after lysing the mitochondria with 0.04% Triton X-100 and repeated freeze-thawing. CS activity was determined spectrophotometrically at 37°C and 412 nm as previously reported [26].

### Histochemistry

LV tissue embedded in OCT was cut into 10 μm cross sections with a cryostat (Thermo Fisher Scientific, Ottawa, ON, CA) maintained at -20°C. Capillary density quantification and cross sectional area (CSA) measurements were adapted from previous work [27, 28]. Briefly, sections were fixed in 10% formalin buffered solution for 10 min, permeabilized with 0.5% TritonX-100 for 10 min, and then blocked in 10% goat serum for 30 min. Sections were incubated overnight in 1.5% goat serum with the appropriate primary antibodies specific for the endothelium (collagen IV, 1:50) and sarcolemma (dystrophin, 1:200) (Developmental Studies Hybridoma Bank, Iowa City, IA, USA). After three 5 min washes in PBS, sections were incubated for 1 hour in 3% goat serum with the appropriate fluorescent secondary antibodies (Life Technologies, Burlington, ON, CA). Nuclear counterstaining was also performed by incubating slides for 5 min in 4',6-diamidino-2-phenylindole (DAPI) prior to visualization. Capillaries were manually counted from 10 separate regions of each cross section (>50 fibres/cross section) and longitudinal fibres were discounted from analysis.

CSA was calculated by outlining all fibres from 10 separate regions of each cross section (>50 per type per muscle per animal). All imaging was performed with an Axio Observer Z1 fluorescent microscope and associated AxioVision software (Carl Zeiss).

To quantify LV fibrosis, sections were stained using picrosirius red as previously reported [29]. Sections were imaged using an Olympus FSX 100 light microscope and images were acquired in Cell Sense software (Olympus, Tokyo, Japan). Using standard light microscopy, picrosirius red staining reveals collagen as red and cardiac fibers and cytoplasm as yellow. To quantify fibrosis, Cell Sense software was used to threshold images, which isolated total area of red (fibrosis) and total area of yellow (cytoplasm/fiber). Fibrosis was expressed as a percent of total tissue area. For each animal, fibrosis was determined by averaging 5 different locations within the LV.

### Statistical Analysis

A one-way ANOVA, followed by a Newman-Keuls Multiple Comparison post-hoc analysis was used to determine the effects ET and HIIT in the setting of HS. A *p<0*.*05* was considered statistically significant. All pre- and post-intervention MAP and HR measures were analyzed for significance using a paired t-test with the α-value set to *P<0*.*05*.

## Results

### Animal phenotype and characterization of hypertension

We first aimed to characterize the phenotype of the hypertensive rats. There were no differences between groups of animals in MAP, HR or body weight prior to commencing the 4-week intervention (Table 2). Similar to previous reports, the LS group displayed mild hypertension [30, 31], while the consumption of the HS diet further increased (P<0.05) resting MAP ~10mmHg (Table 2), confirming the induction of overt hypertension in these animals.

**Table 2:** Morphometrics and hemodynamics of LS-SED, HS-SED, HS-ET and HS-HIIT rats before and after 4 weeks of training.

		Baseline				4 weeks			
Parameter		LS-SED	HS-SED	HS-ET	HS-HIIT	LS-SED	HS-SED	HS-ET	HS-HIIT
**Mean Arterial Pressure**	(mmHg)	134 ± 2	135 ± 18	129 ± 2	137 ± 3	154 ± 3* †	163 ± 1*	165 ± 3*	165 ± 2*
**Heart rate**	(bpm)	398 ± 15	417 ± 16	410 ± 10	420 ± 5	396 ± 14	358 ± 11*	364 ± 8*	369 ± 11*
**Body weight**	(BW, g)	235 ± 7	245 ± 4	235 ± 6	245 ± 4	324 ± 6*	320 ± 5*	305 ± 7*	316 ± 4*
**Heart weight**	(HW, g)	-	-	-	-	0.91 ± 0.02	0.95 ± 0.01	0.94 ± 0.03	1.14± ± 0.03†
**Tibia length**	(cm)	-	-	-	-	4.15 ± 0.02	4.15 ± 0.02	4.2 ± 0.03	4.13 ± 0.02
**HW/BW**	(mg/g)	-	-	-	-	2.8 ± 0.08	2.9 ± 0.08	3.1 ± 0.09	3.6 ± 0.1

Low sodium sedentary (LS-SED), high sodium SED (HS-SED), HS endurance training (HS-ET) and HS high intensity interval training (HS-HIIT).

* vs. baseline;

† vs. all other groups; *P<0*.*05*. Data are means ± SEM.

Resting MAP was not altered by either 4 weeks of ET or HIIT (Table 2). A loss of body weight represents an early clinical marker of HF in these animals, however body weight was unaffected by either the HS diet or exercise training (Table 2), confirming the absence of pronounced clinical symptoms of late stage HF. HR and tibia length remained consistent between groups (Table 2).

**Fig 1:**
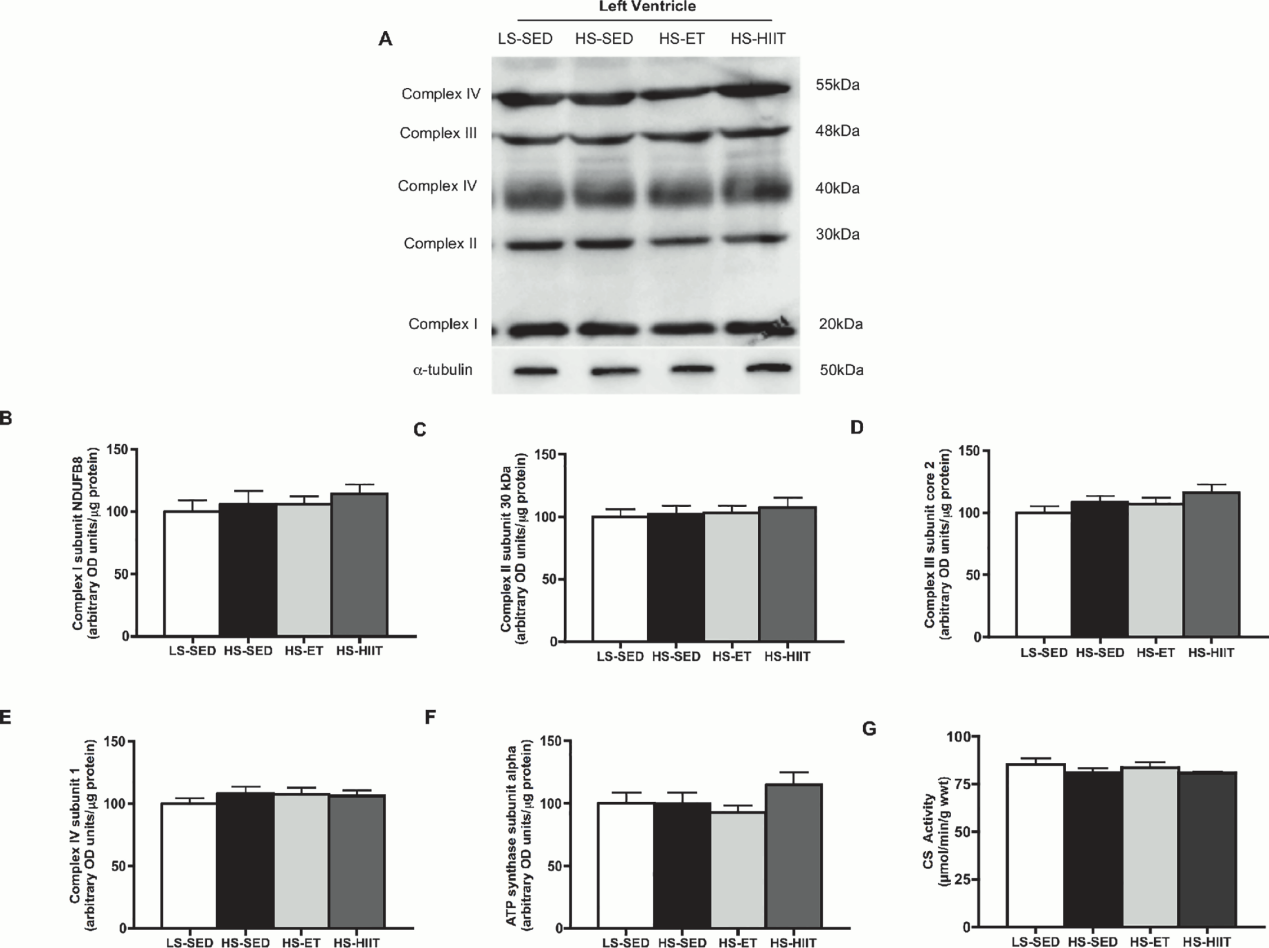
Mitochondrial content and citrate synthase (CS) activity in the left ventricle (LV). A. Representative western blot of LV OXPHOS proteins in low sodium sedentary (LS-SED), high sodium SED (HS-SED), HS endurance training (HS-ET) and HS high intensity interval training (HS-HIIT) B-E. Density quantifications of LV OXPHOS in LS-SED, HS-SED, HS-ET and HS-HIIT, demonstrating no change in mitochondrial content in both ET and HIIT. F. CS activity as expressed per gram wet weight. HS had no effect on CS activity. Data are means ± SEM.

As mitochondrial content is reduced in end stage HF, we investigated the content of 5 OXPHOS proteins and CS activity. After 4 weeks of HS, OXPHOS protein content and CS activity were not altered, suggesting the absence of reductions in mitochondrial content (Fig. 1). Altogether, these data confirmed that after 4 weeks of consuming HS, the Dahl/SS rat begins to display hypertension, as opposed to end stage HF, and therefore represents an ideal model/age to determine the effect of exercise intensity on the molecular adaptations in the heart in the early stages of HF progression.

### Effect of training on fibrosis

**Fig 2:**
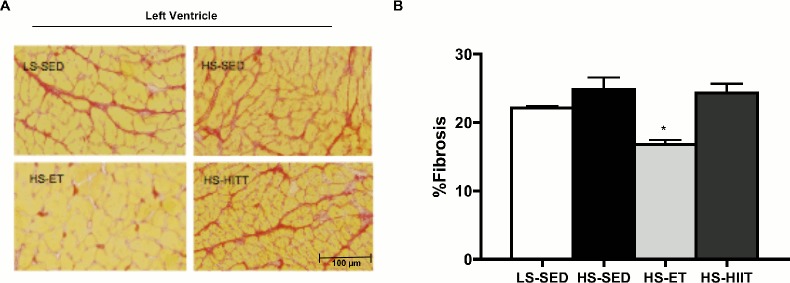
Left ventricular (LV) fibrosis in response to high sodium (HS) with endurance training (ET) and high intensity interval training (HIIT). A. Composite wide field microscopy images of LV; with picrosirius red stain for fibrosis; low sodium-SED (LS-SED; top left), HS-SED (top right), HS-ET (bottom left) and HS-HIIT (bottom right). B. Quantification (% area) of fibrosis; HS-ET demonstrates significantly less % area of fibrosis vs. all other groups: * vs. LS-SED, HS-SED and HS-HIIT, *P<0*.*05* C. Data are means ± SEM.

We examined myocardial fibrosis to determine if ET and HIIT were effective at reducing the development of fibrosis in Dahl/SS rats. While the consumption of HS did not further increase fibrosis as compared to LS, ET training reduced (P<0.05) fibrosis by ~40% (Fig. 2). In contrast to ET, HIIT did not ameliorate the development of fibrosis (Fig. 2). These data suggested that ET preferentially protects the heart from the development fibrosis in response to hypertension.

### The effects of training on hypertrophy and markers of HF

**Fig 3:**
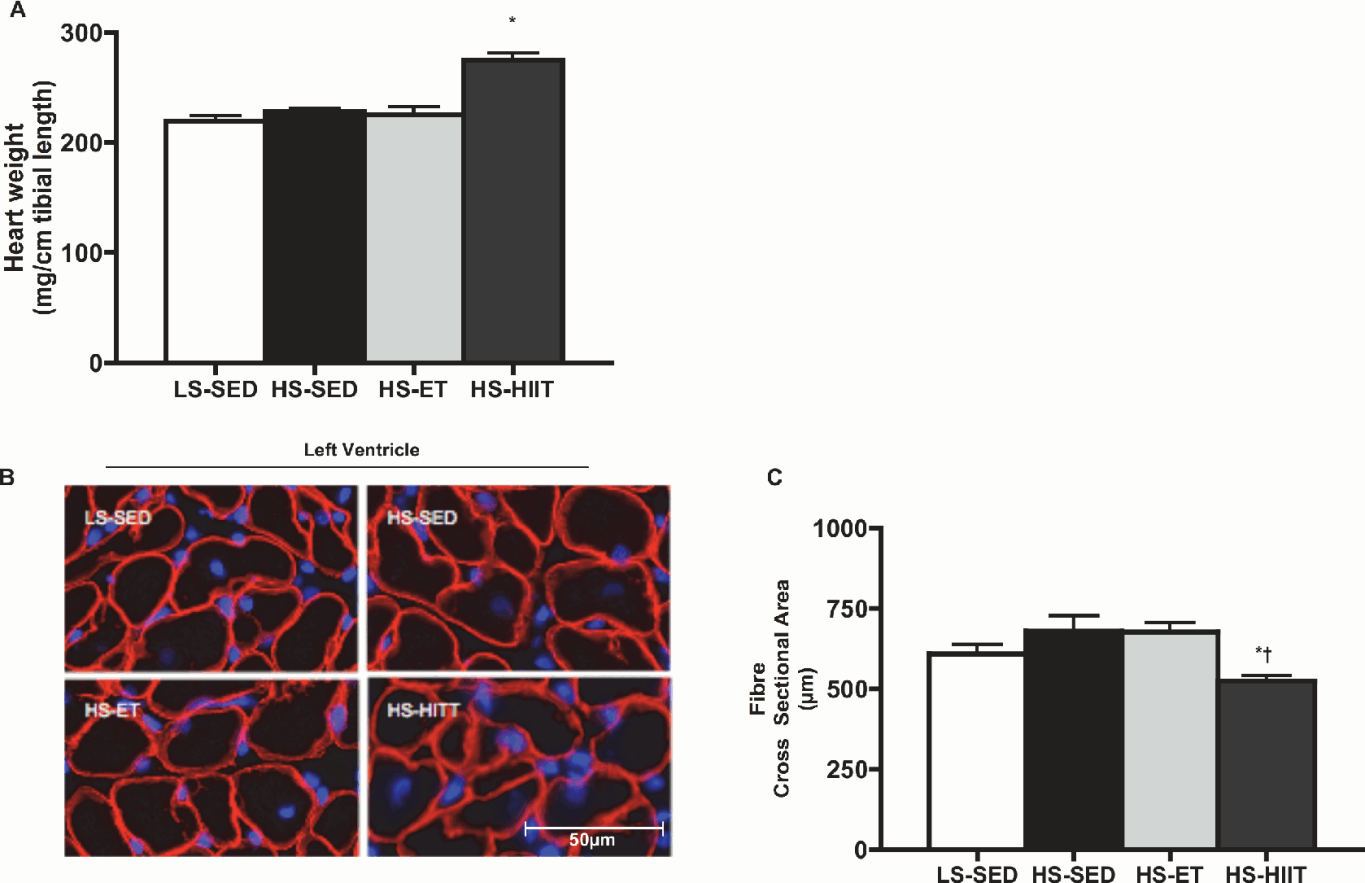
Effect of high sodium (HS) with endurance training (ET) and high intensity interval training (HIIT) on heart weight and cardiac fibre cross-sectional area. A. Heart weight normalized to tibia length (HW/TL) in low sodium (LS), HS-ET and HS-HIIT. HS-HIIT demonstrates a significant increase in HW/TL when compared to all other groups: * vs. LS-SED, *P<0*.*05*. B. Composite wide field microscopy images of left ventricle; LS-SED (top left), HS-SED (top right), HS-ET (bottom left) and HS-HIIT (bottom right). C. Left ventricle cardiac fibre cross-sectional area in LS-SED, HS-SED, HS-ET and HS-HIIT. HS-HIIT demonstrates a decrease in cross-sectional area when compared to HS-SED and HS-ET: * vs. HS-SED, vs. **†** HS-ET; *P<0*.*05*. Data are means ± SEM.

Consumption of a HS diet by Dahl/SS rats induces cardiac hypertrophy after 11 weeks [22], however in the current study, 4 weeks of HS was not sufficient to induce significant hypertrophy in the sedentary group (Table 2 and Fig. 3A). Also, ET training did not affect heart weight. Surprisingly however, HIIT increased heart weight by ~20% (Table 2 and Fig. 3A) demonstrating that HIIT initiated the development of cardiac hypertrophy. Similar to cardiac weight, HS and ET did not affect CSA (Fig. 3B and C). In contrast, HIIT resulted in an ~20% reduction in CSA (Fig. 3B and C), indicative of alterations in cardiac fibres and potential for pathological remodeling.

**Fig 4:**
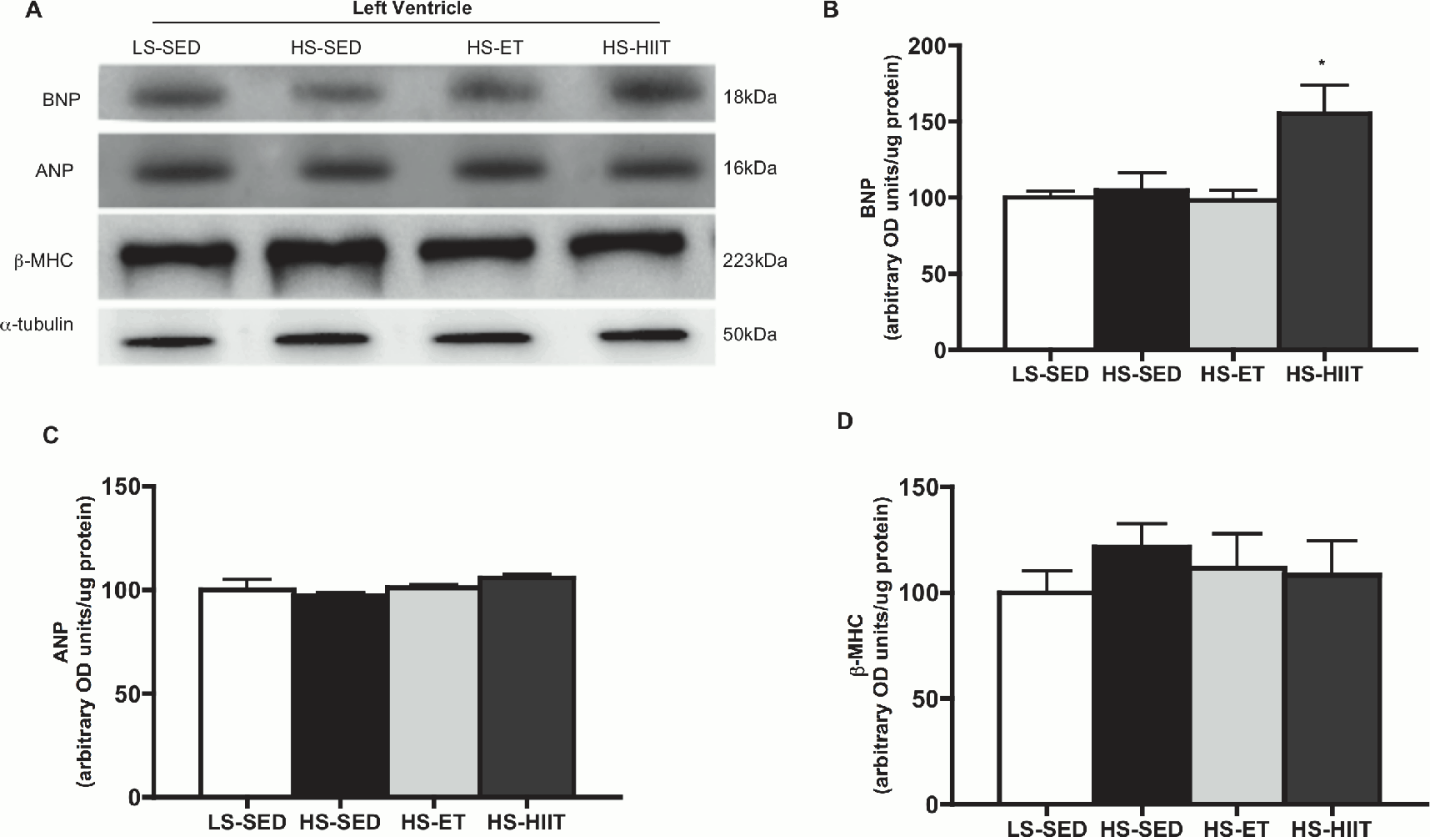
Western blot analysis of brain natriuretic peptide (BNP), atrial natriuretic peptide (ANP) and β-myosin heavy chain (β-MHC). A. Representative blots; α-tubulin is presented as a loading control. B. Density quantifications of BNP of low sodium sedentary (LS-SED), high sodium SED (HS-SED), HS endurance training (HS-ET) and HS high intensity interval training (HS-HIIT), demonstrating an decrease in protein as a result of HS-HIIT; • vs. LS-SED, HS-SED and HS-ET; *P<0*.*05*. C. Density quantifications of ANP protein. D. Density quantifications of protein. Data are means ± SEM.

To further investigate the potential that HIIT exacerbated the development of HF, we determined the protein expression of BNP, ANP and β-MHC (Fig. 4). HIIT animals demonstrated ~50% higher BNP content (P<0.05) when compared to LS-SED, HS-SED and HS-ET suggesting a progression towards HF (Fig. 4B). ANP content was unchanged in response to HS or exercise (Fig. 4C). β-MHC content remained unchanged in all groups (Fig. 4D).

**Fig 5:**
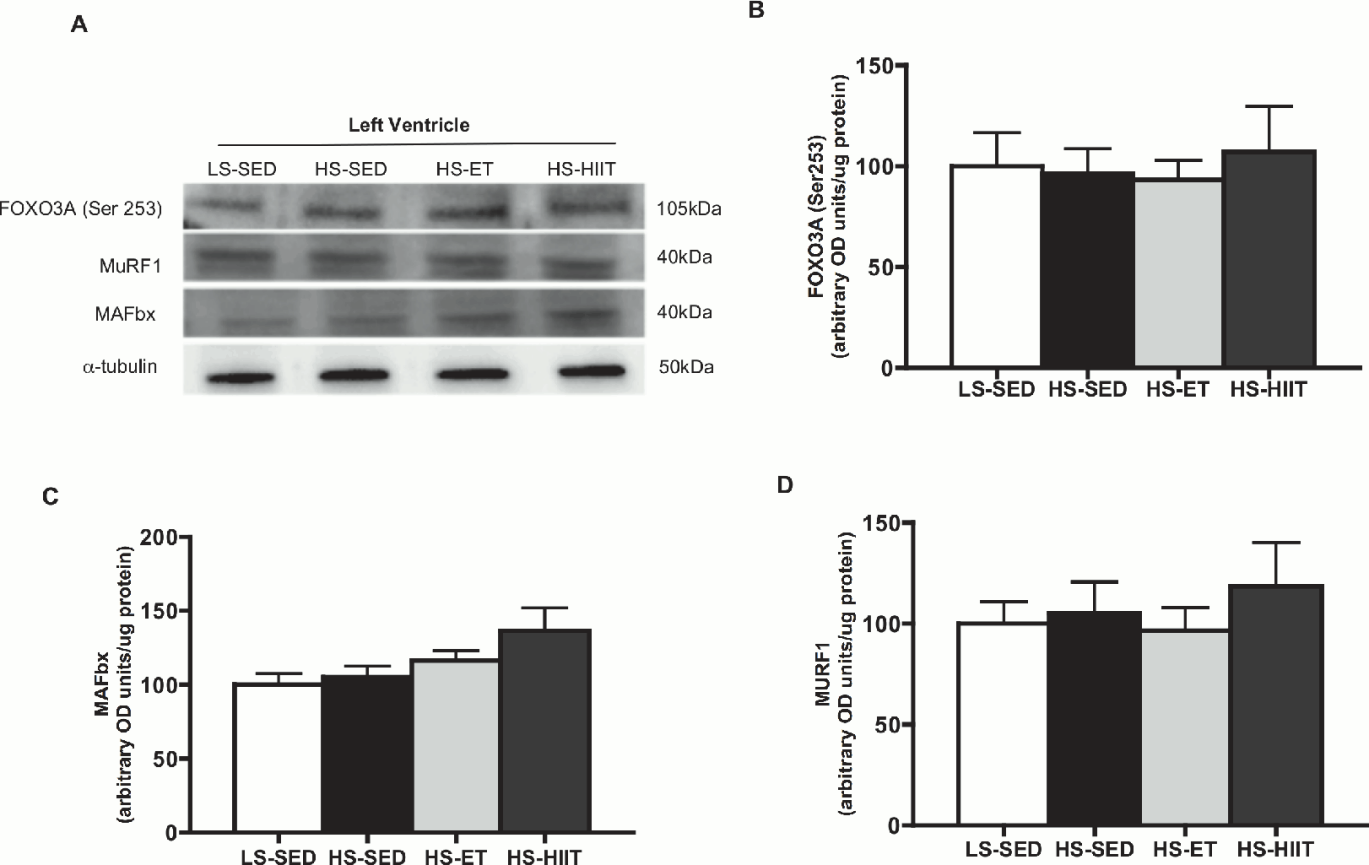
Western blot analysis for FOXO3a (Ser253), MuRF1 and MAFbx. A. Representative blots; α-tubulin is presented as a loading control. B. Density quantifications of FOXO3a (Ser253) protein in LV of low sodium sedentary (LS-SED), high sodium SED (HS-SED), HS endurance training (HS-ET) and HS high intensity interval training (HS-HIIT). C. Density quantifications of MAFbx. D. Density quantifications of MuRF1 protein. Data are means ± SEM.

Molecular regulators of muscle hypertrophy and atrophy have also been linked to the transition from hypertrophy to HF [32, 33], however, we examined several proteins associated with atrophy, and show that FOXO3A, MuRF1 and MAFbx were not altered in any condition (Fig. 5A-D).

Altogether, these data suggest that while ET did not elicit a hypertrophic response, HIIT induced cardiac remodeling associated with hypertrophy in the presence of elevated BNP levels indicating that the remodeling that occurred in response to HIIT was pathological.

### Effects of training on cardiac capillarization

**Fig 6:**
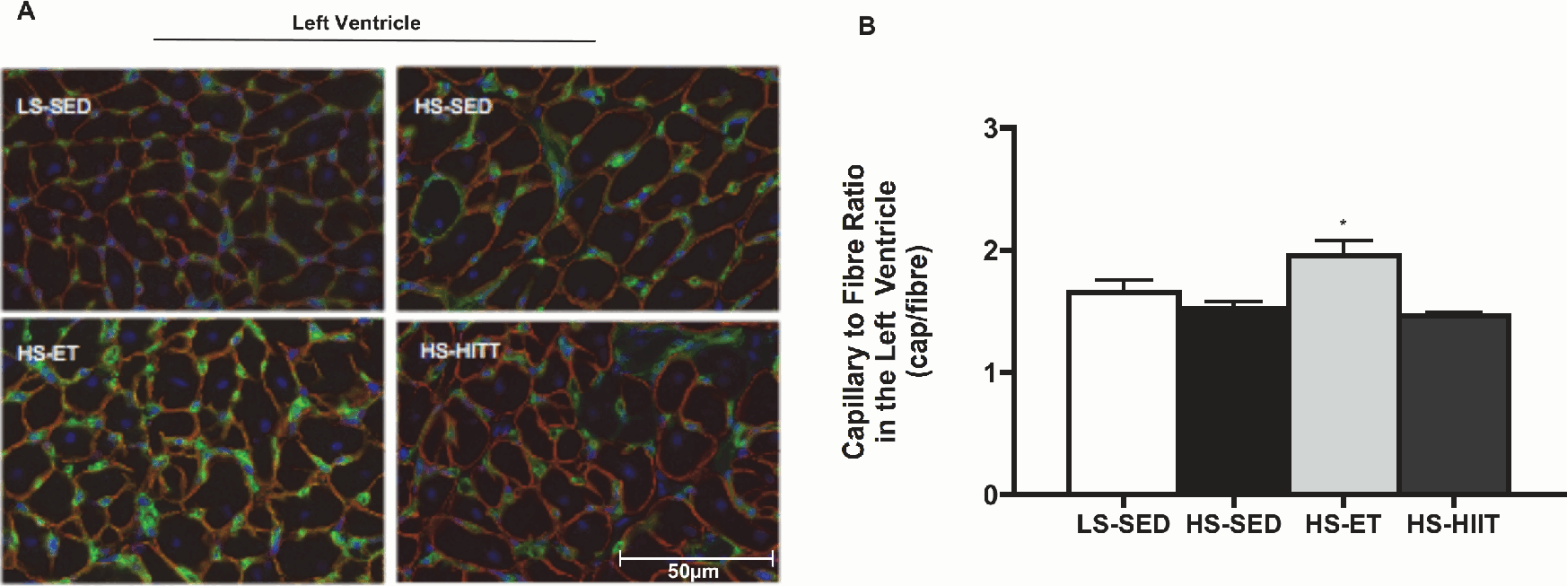
Left ventricular (LV) capillary to fibre ratio. A. Composite wide field microscopy images of LV; of low sodium sedentary (LS-SED-top left), high sodium SED (HS-SED-top right), HS endurance training (HS-ET-bottom left) and HS high intensity interval training (HS-HIIT-bottom right) B. LV capillary to fibre ratio demonstrating a significant increase in response to HS-ET; * vs. LS-SED, HS-SED and HS-HIIT; *P<0*.*05*. Data are means ± SEM.

**Fig 7:**
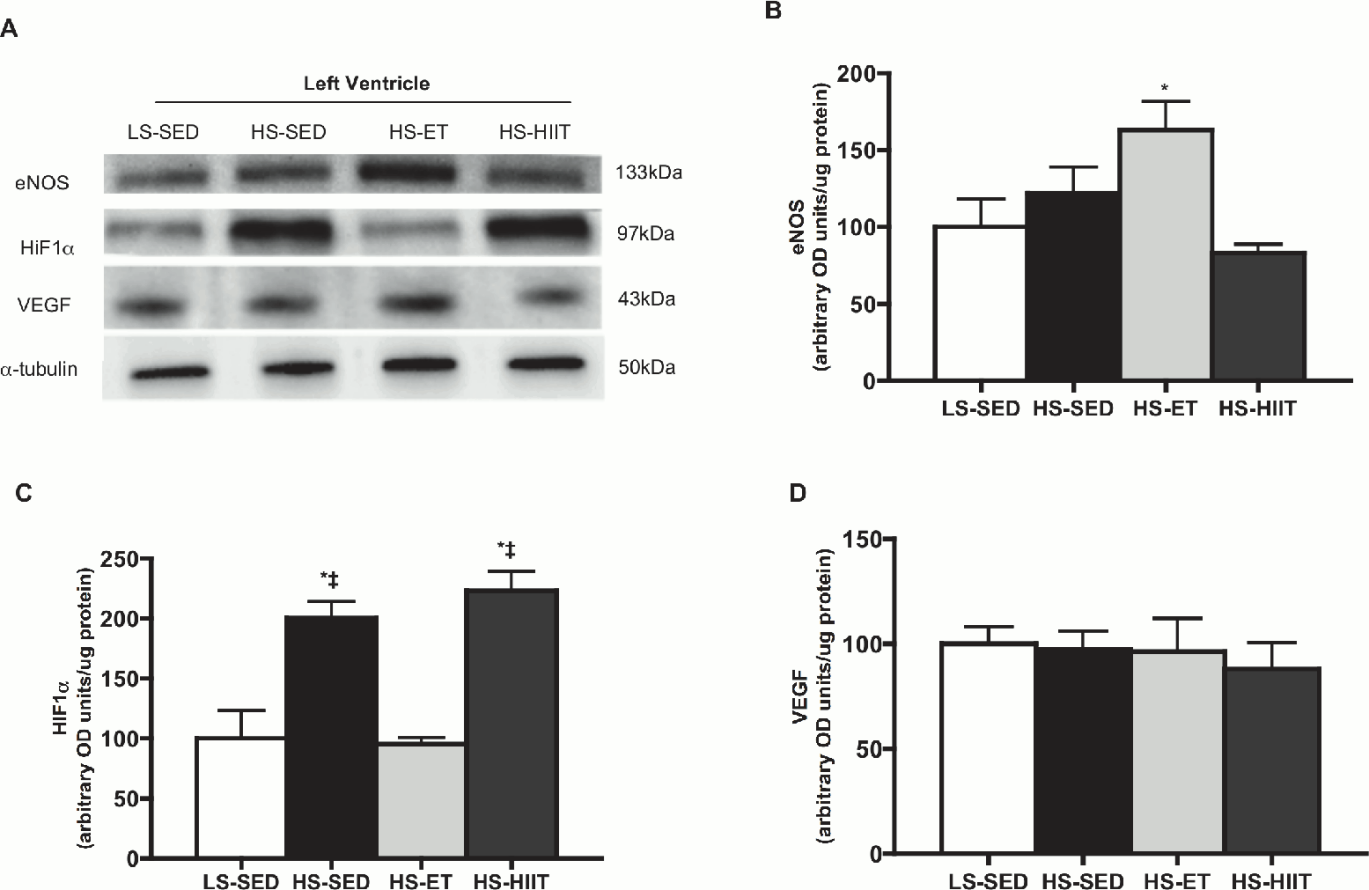
Western blot analysis of eNOS, HIF1α, and VEGF. A. Representative blots; α-tubulin is presented as a loading control. B. Density quantifications of eNOS protein in LV of low sodium sedentary (LS-SED), high sodium SED (HS-SED), HS endurance training (HS-ET) and HS high intensity interval training (HS-HIIT), demonstrating a significant increase as a result of ET, * vs. all other groups; *P<0*.*05* C. Density quantifications of HIF1α, demonstrating a significant increase in response to both HS and HS-HIIT; * vs. LS-SED; ‡ vs. HS-ET. D. Density quantifications of VEGF protein. Data are means ± SEM.

While HS did not reduce capillary/fibre ratios at this early stage of disease progression, animals that underwent ET had a higher (P<0.05) capillary/fibre ratio by ~20%, while in contrast HIIT had no effect (Fig. 6A and B). To further support these data, we examined the protein expression of known angiogenic factors (Fig. 7). Specifically, we found that ET increased (P<0.05) the protein content of eNOS within the heart (Fig. 7B). In addition, HIF1α, a protein responsive to hypoxia, was elevated in both HS-SED and HS-HIIT groups (Fig. 7C). In contrast, ET training, and the resulting angiogenesis, may have contributed to the decreased expression of HIF1α because ET reduces myocardial oxygen demand, and therefore would lower hypoxic stimuli (Fig. 7C). VEGF protein was not altered in any group (Fig. 7D). Taken together, the current data indicated that ET, but not HIIT, induced angiogenesis within the LV of Dahl/SS rats.

## Discussion

The current data provides evidence, that before overt HF develops in Dahl/SS rats fed a HS diet, classical ET: i) ameliorated fibrosis, and ii) induced coronary angiogenesis. In stark contrast, HIIT induced: i) LV hypertrophy, ii) a reduction in cardiac fibre CSA, and iii) increased the expression of BNP, a protein associated with HF, without altering fibrosis or angiogenesis. Altogether, the current data suggested that ET and HIIT induce divergent signals in the heart, and the potential that HIIT exacerbated the development towards HF in hypertensive Dahl/SS rats.

### Left ventricular fibrosis

Cardiac remodeling, and in particular an increase in fibrosis, is associated with diastolic dysfunction [22, 34]. In the current study, significant fibrosis was apparent in the LV of LS and HS fed rats, with the exception of ET trained rats which displayed ~40% less fibrosis. This indicated that ET, but not HIIT, prevented the development of fibrosis in the Dahl/SS rats. The relationship between fibrosis and diastolic dysfunction has long been defined, and many studies have successfully reduced diastolic stiffness by targeting the signaling factors involved in the development of fibrosis, such as transforming growth factor-β, the sodium-hydrogen exchanger, angiotensin II receptor-mediating signaling, and chymase [35–37]. HF manifestation in the Dahl/SS rat has been largely characterized as diastolic dysfunction, and fibrosis is considered to be a mediator of this process. Interventions such as exercise and pharmacological compounds (e.g., resveratrol, angiotensin receptor blockers) reduce the formation of fibrosis and are associated with improved diastolic function in Dahl/SS rats [38, 50] and other animal models [22, 38, 39]. Therefore, the current data suggests that ET promoted a phenotype that would improve LV relaxation, decrease end diastolic pressure and improve overall filing, which are characteristics of improved diastolic function. The signals that have elicited the divergent results in fibrosis between ET and HIIT remain unclear.

While alterations in blood pressure could represent a potential explanation, in the current model, neither ET nor HIIT altered resting blood pressure (Table 2). However, it is unclear how blood pressure was altered in these animals during or immediately after exercise. Regardless, our results indicate that only ET prevented fibrosis, data that is supported by previous findings that ET protects against, and reverses, the development of fibrosis in Dahl/SS rats in the later stages of disease progression [40]. Therefore, with respect to fibrosis, ET represented the optimal exercise intervention in presence of hypertension.

### Cardiac hypertrophy and cardiac fibre cross-sectional area

Pressure-induced cardiac remodeling is initially associated with concentric cardiac hypertrophy that is beneficial. While HS did not cause hypertrophy, HIIT elicited both LV hypertrophy and a reduction in cardiac fibre CSA, responses not observed in ET animals. Cardiac hypertrophy occurs in response to various stimuli such as chronic exercise training (volume overload) and hypertension (pressure overload), and the hypertrophy that develops is specific to the stimuli that caused the increase in LV mass. Physiological hypertrophy is characterized by normal organization of cardiac structure and normal or enhanced cardiac function. In contrast, pathological hypertrophy is commonly associated with the upregulation of fetal genes (e.g., β-MHC) [40, 41], fibrosis and cardiac dysfunction and is recognized as an independent risk factor for morbidity and mortality [42–45]. Early progression to HF involves the development of hypertrophy and a reorganization of cardiomyocytes. The etiology of the reduction of CSA in the HIIT animals remains unclear, and was an unexpected finding. However, given the various stages of remodeling as a result of hypertension, this may be due the timing of our study. The animals were hypertensive for 4 weeks, and to our knowledge there are no studies that have investigated the adaptations occurring in the cardiomyocytes at this early time point.

In the setting of disease, initially myocardial hypertrophy is a compensatory mechanism, by which the LV adapts to an increased systolic load (e.g., hypertension, aortic stenosis), with the aim of normalizing/restoring LV wall stress and the maintenance of cardiac output [46]. While in the early stages of disease progression, HS did not cause hypertrophy in the SED or ET animals, while HIIT caused significant hypertrophy to develop. This strongly supports the notion that in the presence of hypertension, HIIT promotes pathological remodeling of the LV.

### Brain and atrial natriuretic peptides and β-myosin heavy chain

Neurohormonal activation is a physiological response and important prognostic marker in patients with chronic HF. Specifically, in HF an elevated BNP level is associated with impaired left ventricular ejection fraction and early mortality [47]. HIIT animals demonstrated significantly higher LV BNP levels when compared to ET or HS alone. In response to increased filling pressure BNP is released primarily from the LV promoting vasodilatation and natriuresis [48]. On the other-hand, ANP is released in response to exercise and remains elevated in the presence of HF, but returns to baseline post-exercise in healthy individuals [49]. Therefore, the fact that we did not see an elevation in ANP is not surprising, as the time point studied represented the early stages of HF progression. Furthermore, we did not detect an increase in β-MHC typically seen in advanced models of HF [40, 41] confirming the absence of overt HF in our animals after 4 weeks of HS. The elevation of BNP in the HIIT group, along with hypertrophy and alterations in CSA, again suggests that HIIT promoted a phenotype that may transition into end-stage HF at an accelerated rate.

### Capillarization, eNOS, and HIF1α content

The balance between cardiac growth and coronary angiogenesis is a key determinant of cardiac function, and disruption of this balance is implicated in the transition from physiological to pathological hypertrophy and HF [50]. In addition to protecting against fibrosis, ET promotes angiogenesis in this model, and an increase in LV capillary/fibre ratio [51]. eNOS is found primarily in the vascular endothelium and a concomitant loss of endothelium and eNOS content is associated with various disease states, such as hypertension, diabetes and HF [52–54]. Endurance exercise is effective at restoring vascular reactivity and eNOS levels in these disease states [19, 55, 56]. Although we did not see a significant reduction in eNOS due to HS at this early time point in the disease progression, ET increased eNOS content, while HIIT did not. This may also have implications for the development of pathological hypertrophy seen in our HIIT animals, as previous studies have demonstrated that nitric oxide (NO) production through eNOS plays an important role in the regulation of cardiac hypertrophy[56]. The studies in which eNOS signaling was augmented by the administration of a calcium antagonist or angiotensin-I converting enzyme inhibitors demonstrated improvements in myocardial remodeling and HF [57, 58].

The elevation of eNOS protein content and increase in capillary/fibre ratio in the ET animals occurred together with a decrease in HIF1α protein content. HIF1α is considered to be the mediator of physiological and patho-physiological responses to hypoxia and exercise training. HIF1α protein content decreases in response to chronic exercise training in both heart and skeletal muscle [59–61]. The physiological mechanisms by which cells respond to hypoxic stimuli are only beginning to be elucidated at the molecular level. However, the role of HIF1α in the activation of VEGF gene transcription in hypoxic cells is well established [59, 62]. Hypoxia, and the molecular responses to hypoxia, play an important role in the pathology of major causes of mortality, such as myocardial ischemia, and chronic heart and lung diseases [59].

The development of myocardial hypertrophy transiently causes hypoxia and HIF1α accumulation, leading to the initiation of angiogenesis [63]. Angiogenesis supports the growth and survival of the newly thickened myocardium. However, as hypertrophy continues to develop, prolonged hypoxia leads to p53 accumulation, which mediates the inactivation of HIF1α and results in eventual cardiac failure due to further inhibition of angiogenesis [63]. The fact that our animals demonstrated hypertrophy in response to HIIT, together with an increase in HIF1α protein, and without a concomitant increase in VEGF protein, suggests that the adaptive molecular signals are in the early stages and prior to compensatory angiogenesis typically seen in myocardial hypertrophy. The lack of response of VEGF protein content was not expected, although it is known that the effect of training on VEGF protein is temporal [64]. Angiogenesis as a result of chronic exercise training is dependent on the complex coordination of the metabolic signals responsible for both positive and negative angiogenic factors. While studies in healthy rodents have shown that interval and moderate exercise training had similar effects on LV capillary density [65], to our knowledge the present data are the first to demonstrate that HIIT does not induce angiogenesis in the hypertrophied LV in the presence of hypertension prior to the development of end stage HF.

The current data support the supposition that, classical ET attenuates cardiac fibrosis, ameliorates pathological hypertrophy, and stimulates angiogenesis in the LV in the presence of hypertension, supporting the longstanding belief in the effectiveness of ET in disease. In contrast, HIIT did not reduce fibrosis or promote angiogenesis. Critically, myocardial weight increased along with an elevated BNP expression as a result of HIIT, all of which are consistent with pathological remodeling and deleterious cardiac function. The negative impact of HIIT on the LV in the presence of hypertension highlights the need for further research on the effects of HIIT in the presence of disease. Altogether, our data demonstrates that ET and HIIT induce divergent molecular signatures in the hearts of hypertensive rats.
